# Establishment and application of a fluorescence-based nucleic acid detection system for canine parvovirus

**DOI:** 10.3389/fvets.2025.1691815

**Published:** 2025-11-19

**Authors:** Shaoting Weng, Yuelong Cui, Shengming Ma, Kaiqi Lian, Mingliang Zhang, Liqiang Wang, Xuekun Dou, Rong Huang, Longfei Zhang, Yao Wang

**Affiliations:** 1 Department of Biotechnology, Anyang Institute of Technology, Anyang, China; 2 Department of Radiotherapy, Anyang District Hospital, Puyang, China; 3 College of Animal Science and Technology, Henan Institute of Science and Technology, Xinxiang, China

**Keywords:** canine parvovirus, VP2 gene, recombinase polymerase amplification, T7 endonuclease I, fluorescent probes, colloidal gold lateral flow assay

## Abstract

Canine Parvovirus (CPV) is a significant pathogen threatening the health of canine worldwide, characterized by high infectivity and fatality rates. A rapid, accurate, and convenient detection method is crucial for early intervention and control of CPV infections. In this study, a novel visual detection method for CPV based on nucleic acid mismatch endonuclease detection (NMED) was established. This method amplifies the conserved region of the CPV VP2 gene through optimized recombinase polymerase amplification (RPA). Subsequently, the amplified products are hybridized with specially designed fluorescently labeled probes. Then, T7 endonuclease I (T7E I) specifically recognizes and cleaves the hybridized products. Finally, the detection signals are visually interpreted using colloidal gold lateral flow assay (LFA). The results of our study indicate that the NMED method can complete DNA sample analysis within 50 min, with strong specificity and no cross - reactions with other common canine viruses. Sensitivity tests show that its detection limit is above 10 copies/μL. In the validation of 35 clinically suspected samples, the overall coincidence rate with RPA and qPCR is over 97.14%, and it reaches 100% in strongly positive samples. In conclusion, this study has established an efficient, specific, and visual nucleic acid detection method for CPV. Its establishment provides an important technical support for the early warning and precise prevention and control of CPV infections.

## Introduction

Canine Parvovirus (CPV) is a highly pathogenic single-stranded DNA virus primarily infecting canids, especially puppies (1). It can cause myocarditis or hemorrhagic enteritis, with an extremely high mortality rate (2). Its genome is approximately 5.2 kb, encoding non-structural proteins (NS1/NS2) and structural proteins (VP1/VP2) (3). Sequence variations in the VP2 gene determine viral subtypes (2a, 2b, 2c), and due to its high conservation, VP2 serves as the core target for detection (4). Currently, there are no specific antiviral drugs for CPV, making early detection crucial for interrupting transmission and improving prognosis (5, 6).

There exists a diverse array of traditional detection technologies for CPV-Based Molecules. Colloidal Gold Immunochromatography (based on VP2 protein monoclonal antibodies) is simple to operate (completed within 10 min), but exhibits low sensitivity (LOD = 10^4^ copies/μL) and insufficient specificity (false positive rate of 12%), making it prone to cross-reactivity (7). qPCR and Multiplex PCR Offer high sensitivity (LOD ≈ 10^2^ copies/μL) and the capability to detect multiple viruses simultaneously. However, they rely on expensive instrumentation (e.g., ABI 7500), require skilled personnel for operation, and multiplex PCR can suffer from false negatives due to primer-primer interactions (8, 9). Magnetic Bead Chemiluminescence Immunoassay Utilizes VP2 antigen-conjugated magnetic beads and acridinium ester-labeled antibodies for signal amplification, achieving a very low LOD of 0.36 ng/mL. It enables quantitative assessment of antibody titers for evaluating vaccine efficacy. Its drawbacks include complex operational procedures and high costs (10). In recent years, isothermal amplification techniques (LAMP, RPA, NASBA) have become central to Point-of-Care Testing (POCT). They Eliminates the need for complex instruments; rapid amplification under mild conditions (39 °C, completed within 15 min) (11–13). When combined with microfluidic chips or quantum dot immunochromatography, LOD reaches 10 copies/μL, reducing costs to 1/5 of traditional methods. However, they are susceptible to reaction condition interference, exhibits lower specificity, and carries risks of contamination and false-positive results (14–16). CRISPR-Based Diagnostics (CRISPR-Dx) leverages the collateral cleavage activity of Cas12a/Cas13a. Activated Cas enzymes cleave non-specific fluorescent probes, releasing a detectable signal. Often combined with RPA transcription and lateral flow assay (LFA) or electrochemical biosensors, enabling detection of viral DNA as low as 100 aM within 30 min (17). Key advantages include signal amplification, convenience, efficiency, and the elimination of expensive instruments. Limitations include high requirements for crRNA design and synthesis; non-specific cleavage by Cas enzymes may lead to off-target effects (18). Enzymatic Mismatch Cleavage (EMC) Utilizes S1 nuclease family enzymes (e.g., T7E1, EndoV, CELI) to recognize and cleave mismatch sites (insertions/deletions, SNPs) in DNA heteroduplexes (19). This makes EMC suitable for variant detection and polymorphism analysis (20). For example, CEL I combined with isoelectric focusing gel electrophoresis can detect 100% of 25 known variants (18 single-base substitutions, 7 small insertions/deletions), offering high efficiency, speed, and low cost (21, 22).

**Figure 1:**
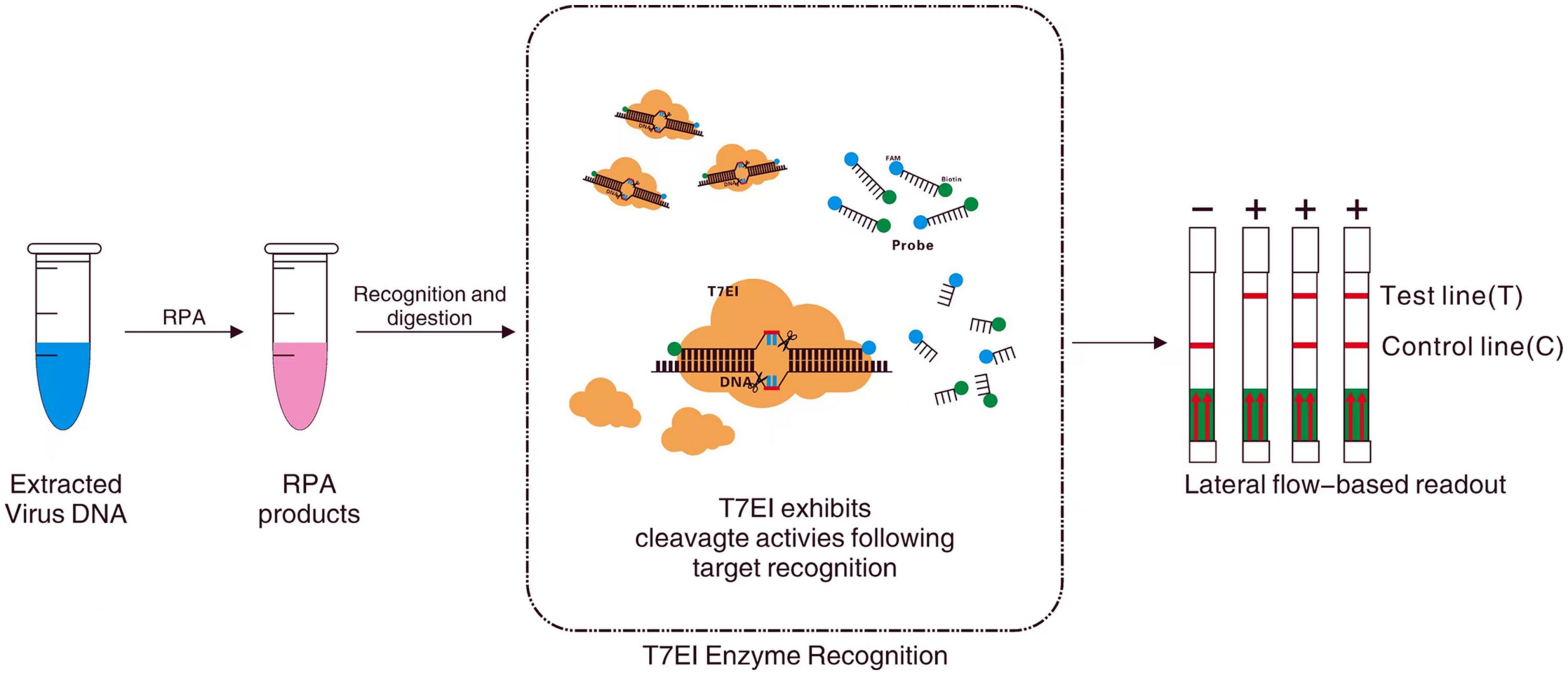
Workfow of NMED assay, which includes RPA amplification, nucleic acid hybridization, enzyme digestion, and LFA. Following nucleic acid lysis, the target sample undergoes Recombinase Polymerase Amplification (RPA), generating a large quantity of DNA containing the target sequence. Subsequently, leveraging the principle of EMC technology, a specific 6-FAM—Bio fluorescently labeled probe (incorporating two mutated bases) is utilized to recognize the target sequence through nucleic acid hybridization. In the event that the probe binds to the target sequence, it activates the endonuclease activity of T7E I at the mismatch site, leading to the cleavage of the fluorescent probe and the release of FAM and Bio fluorophores. Ultimately, a red line appearing at the test line of the visual LFA indicates a positive result. Conversely, if the probe fails to recognize the target sequence, T7E I is unable to cleave, and the FAM and Bio fluorophores within the probe molecule remain unseparated. Consequently, the absence of a red line at the test line of the visual LFA signifies a negative result.

Traditional CPV detection technologies face limitations in sensitivity, specificity, or practicality. Novel technologies, exemplified by isothermal amplification (e.g., RPA) and CRISPR-Dx, are emerging as core directions for POCT by simplifying operation, enhancing sensitivity, and reducing costs. However, challenges such as insufficient specificity, contamination risks, and the complexity of crRNA design remain for these new technologies. This study employs Nucleic acid Mismatch Endonuclease Detection (NMED) technology for CPV detection (Figure 1), with the aim of developing and optimizing a nucleic acid detection method that combines rapidity, high sensitivity, strong specificity, andvisualizable result acquisition. This work will provide a novel and practical technique for the on-site rapid screening and control of CPV.

## Materials and methods

### Reagents

E.Z.N.A.™ EndoFree Plasmid Midi Kit, PAGE Gel Preparation Kit were purchased from Shanghai Solarbio Biotechnology Co., Ltd.; TwistAmpTM Basic Kit was purchased from TwistDx Bioscience Co., Ltd.; StarSpin Fast Virus DNA/RNA Kit was purchased from Beijing Genstar Biotechnology Co., Ltd.; 1 × Phosphate Buffered Saline, Tryptone, Yeast, Sodium chloride, Ampicillin sodium, Agarose, General DNA Purification, SerBlue nucleic acid dye and Recovery Kit were purchased from Wuhan Servicebio Co., Ltd.; HiScript III 1st Strand cDNA Synthesis Kit, DNA Marker (DL500, DL2000), 6 × DNA Loading Buffer, AceQ Universal U + Probe Master Mix V2, RoomTemp Sample Lysis Kit were purchased from Nanjing Vazyme Biotechnology Co., Ltd. T7E I was purchased from Beijing viewsolid biotechnology Co., Ltd.; DNA Gel Extraction Kit was purchased from Beijing Tsingke Biotech Co., Ltd.

### Primer and probe synthesis

This study was predicated on the VP2 gene sequence of the prevalent strain of Canine Parvovirus (CPV) retrieved from the GenBank database (GenBank Accession No. KF803636.1). A molecular system was constructed using bioinformatics methodologies. Initially, the MegAlign module within the DNASTAR software package was employed to perform a multiple sequence alignment of the target VP2 gene sequence against the corresponding VP2 gene sequences of the representative strains of three CPV subtypes: CPV-2A (M38245), CPV-2B (M24003), and CPV-2C (AF543652). Through homology analysis, highly conserved regions were identified, characterized by 100% nucleotide identity. Subsequently, based on these conserved segments, the Oligo7 primer design software was utilized with stringent parameters to design specific Recombinase Polymerase Amplification (RPA)/quantitative Polymerase Chain Reaction (qPCR) primers. To address the requirement for optimized detection specificity, fluorescent probes with gradient lengths (25–40 nucleotides) were constructed for each target site. The 5′ end of each probe was labeled with the FAM (6-Carboxyfluorescein) fluorophore, while the 3′ end was modified with biotin (Bio) to enable solid-phase coupling. Additionally, two mismatched bases were strategically introduced in the middle section of each probe. All oligonucleotides, including the primers and probes, were synthesized by Sangon Biotech (Shanghai) Co., Ltd. The final oligonucleotide products were reconstituted in TE buffer to prepare working solutions with a concentration of 10 μM. These working solutions were then aliquoted and stored at −20 °C in the dark to prevent degradation caused by repeated freeze–thaw cycles. The detailed experimental parameters and characteristics of the primers and probes are presented in Table 1.

**Table 1:** Primer and probe nucleotide sequences.

Name	Sequences (5′-3′)
RPA-P1-565-F	GCTATGAGATCTGAGACATTGGGTTTTTAT
RPA-P1-790-R	CTGTAGCAAATTCATCACCTGTTCTTAGTA
RPA-P2-547-F	ATGCCATTTACTCCAGCAGCTATGAGATCT
RPA-P2-801-R	ATAAAATGTTCCTGTAGCAAATTCATCACC
RPA-P3-539-F	ATAATACTATGCCATTTACTCCAGCAGCTA
RPA-P3-809-R	CAATCAAAATAAAATGTTCCTGTAGCAAAT
qPCR-547-F	ATGCCATTTACTCCAGCAGCTA
qPCR-691-R	TTGTTGGTGTGCCACTAGTTC
Probe-25	ATACTGGAACTAGTG**CT**ACACCAAC
Probe-30	ATCTCATACTGGAACTAGTG**CT**ACACCAAC
Probe-35	ATACCATCTCATACTGGAACTAGTG**CT**ACACCAAC
Probe-40	CATTAATACCATCTCATACTGGAACTAGTG**CT**ACACCAAC
qPCR-Probe	CAGCTATGAGATCTGAGACATTGG

### Standard virus acquisition and CPV clinical sample preparation

Viral nucleic acids from CPV, Canine distemper virus (CDV), Canine parainfluenza virus (CPIV) and Canine Corona Virus (CCV) were extracted using the StarSpin Rapid Viral DNA/RNA Extraction Kit. 2 μg total RNA of viruse underwent reverse transcription using the HiScript III 1st Strand cDNA Synthesis Kit. Concurrently, DNA was extracted from 35 CPV-suspected samples using lysis buffer. All nucleic acid products were aliquoted into nuclease-free microcentrifuge tubes and stored at −20 °C for subsequent use.

### Plasmid extraction and standard preparation

500 μL of Top10 *E. coli* transformed with the laboratory-stored recombinant plasmid pcDNA3.1-VP2 were inoculated into 50 mL of LB liquid medium supplemented with 100 μg/mL ampicillin. The culture was incubated at 37 °C in a constant-temperature shaker incubator with shaking at 220 rpm for 10 h. Subsequently, the bacterial cells were harvested by centrifugation at 3500 × g for 10 min at room temperature to obtain a cell pellet. Endotoxin-free plasmid DNA was extracted using the E.Z.N.A.™ EndoFree Plasmid Midi Kit, strictly following the standardized protocol provided with the kit. The concentration of the extracted plasmid was determined using a NanoDrop 2000 spectrophotometer and measured 866 ng/μL, with an A₂₆₀/A₂₈₀ ratio of 1.85. The plasmid DNA length was 7,176 bp. The plasmid copy number was calculated using the formula: copies/μL = (6.02 × 10^23^) × (concentration in ng/μL × 10^−9^)/(DNA length in bp × 660). The calculation yielded an original concentration equivalent to 1.1 × 10^11^ copies/μL. This stock solution was serially diluted with sterile TE buffer to prepare a positive standard solution with a final concentration of 1 × 10^7^ copies/μL. The standard solution was aliquoted and stored at −80 °C for future use (23).

### Establishment of RPA amplification system

A 47.5 μL reaction mixture was precisely prepared in a 1.5 mL microcentrifuge tube. 29.5 μL of Free Rehydration buffer was added as the reaction matrix, followed by the addition of 2.4 μL each of the 10 μM forward primer and reverse primer. Subsequently, 1 μL of plasmid DNA template or test sample at a concentration of 20 ng/μL was added. The mixture was brought to the final volume of 47.5 μL using nuclease-free water. After thorough mixing by vortexing and brief centrifugation, the reaction mixture was transferred to a TwistAmp basic reaction tube. The reaction was initiated by adding 2.5 μL of 280 mM MgOAc, followed by thorough mixing. The mixture was then incubated at 39 °C for 20 min. Following the reaction, the product was subjected to 1% agarose gel electrophoresis to confirm amplification efficiency.

### Nucleic acid hybridization and enzymatic digestion reaction

200 ng of the RPA amplification products were mixed with 10 × T7E I buffer (containing 50 mM KCl, 10 mM Tris–HCl, pH 8.0)and a CPV-specific probe (molar ratio of probe to RPA amplification products of 5:1). Nucleic acid hybridization was achieved using a gradient temperature control program: incubation at 95 °C for 5 min, cooling from 95 °C to 85 °C at a rate of 2 °C per second, followed by cooling from 85 °C to 25 °C at 0.1 °C per second, and finally maintaining at 12 °C. Subsequently, 1 μL of T7E I was added, followed by isothermal incubation at 37 °C for 20 min to enable specific cleavage of imperfectly matched hybrid duplexes by the endonuclease.

### Polyacrylamide gel electrophoresis analysis

To prepare a 30% acrylamide gel with an acrylamide-to-bisacrylamide ratio of 19:1, follow these steps: Take 1.5 mL of acrylamide stock solution and successively add 1 mL of 5 × TBE buffer, 85 μL of 10% APS, and 3.8 μL of TEMED. Dilute the mixture to a final volume of 4 mL with nuclease-free water, then insert the comb and allow polymerization at room temperature for 30 min. Once polymerized, remove the comb to form sample wells, add the samples, and fill the electrophoresis tank with 1 × TBE buffer. Perform electrophoresis at a constant voltage of 100 V for 40 min. After electrophoresis, carefully remove the gel to a staining tray containing a solution prepared with 10 × SYBR Green I. Incubate the gel with gentle orbital shaking at room temperature for 1 h to allow dye binding. Following staining, transfer the gel to an imaging system (FIRE READER XS D-55-26. M, UVITEC Ltd., UK), visualize the DNA bands under ultraviolet (UV) light.

### Real-time PCR detection

Highly sensitive detection of CPV DNA was performed using the AceQ Universal U + Probe Master Mix V2 (Vazyme) premix reagent system on a Life Technologies QuantStudio 6 Flex Real-Time PCR System. The experiment strictly adhered to standard probe-based qPCR protocols. The 20 μL single-tube reaction mixture contained: 10 μL of 2 × AceQ Universal U + Probe Master Mix V2 (pre-optimized with ROX passive reference dye for effective correction of well-to-well fluorescence fluctuations), 0.4 μL of qPCR-547-F (10 μM), 0.4 μL of qPCR-691-R (10 μM), 0.2 μL of TaqMan probe (10 μM), and 1 μL of DNA template (sequences provided in Table 1). Nuclease-free water was added to bring the total volume to 20 μL.

The thermal cycling protocol consisted of: Initial Step: 37 °C for 2 min (to digest potential contaminants in the template). Denaturation/Enzyme Activation: 95 °C for 5 min (for complete template denaturation and hot-start enzyme activation). Amplification: 45 cycles of: 95 °C for 10 s (denaturation), 60 °C for 30 s (annealing/extension), with fluorescence acquisition during this step. Final Hold: 12 °C (reaction termination). The positive threshold set at Ct ≤ 30. The nucleic acid concentrations of all samples were calculated using the standard curve equation (linear range: R^2^ ≥ 0.98). A positive control (the DNA of CPV) was included in each run.

### Visual inspection analysis

This study established an immunochromatographic detection method based on the FAM—Bio dual-labeled reporter system. In negative samples, the colloidal gold-labeled detection antibody forms a stable complex with the intact FAM-Bio reporter gene. During chromatography, this complex is captured by the immobilized anti-FAM monoclonal antibody, resulting in a clearly visible red band at the control line (C line). In positive samples, the T7E1 enzyme specifically cleaves the reporter gene, exposing Bio. This allows colloidal gold-labeled streptavidin to bind with high affinity to the free biotin, leading to significant accumulation at the test line (T line), while the control line signal is correspondingly attenuated. This signal transduction mechanism, driven by the molecular cleavage event and combined with the dual-indicator (C line and T line), enables semiquantitative analysis of results based on the color difference between the bands. In semiquantitative assays, the ratio of the detected signal peak area for the Test line (T_Area) to the Control line (C_Area), obtained via an optical sensor, is defined as the Detection ratio (Dr value). The interpretation thresholds for the Dr. value are as follows: Negative is defined as Dr. ≤ 0.05; Positive is defined as Dr. > 0.05; Weak Positive is defined as 0.05 < Dr. ≤ 0.2 (Colloidal Gold Reader GY-670, Henan Guanyu Instrument Co., Ltd.).

### Statistical analysis

Quantitative Assessment of T7E I Cleavage Efficiency Using ImageJ Image Analysis System. The cleavage efficiency of T7E I was quantitatively evaluated based on the ImageJ analysis system. Following preprocessing via 8-bit grayscale conversion, gel bands were standardized using the Gel Analysis module. The Magic Wand tool was employed to precisely determine band area values (Area). Grayscale data from target bands and cleaved bands were collectively processed and converted into cleavage efficiency percentages. All statistical analyses were performed using GraphPad Prism 6.01. Data visualization utilized composite box-and-whisker plots with column bars. Quantitative data are presented as mean ± standard deviation (SD). The experimental design strictly adhered to the use of three independent biological replicates. Each replicate experiment included technical replicates and negative controls to ensure the reliability and reproducibility of the experimental results.

## Results

### Screening of CPV RPA primers

**Figure 2:**
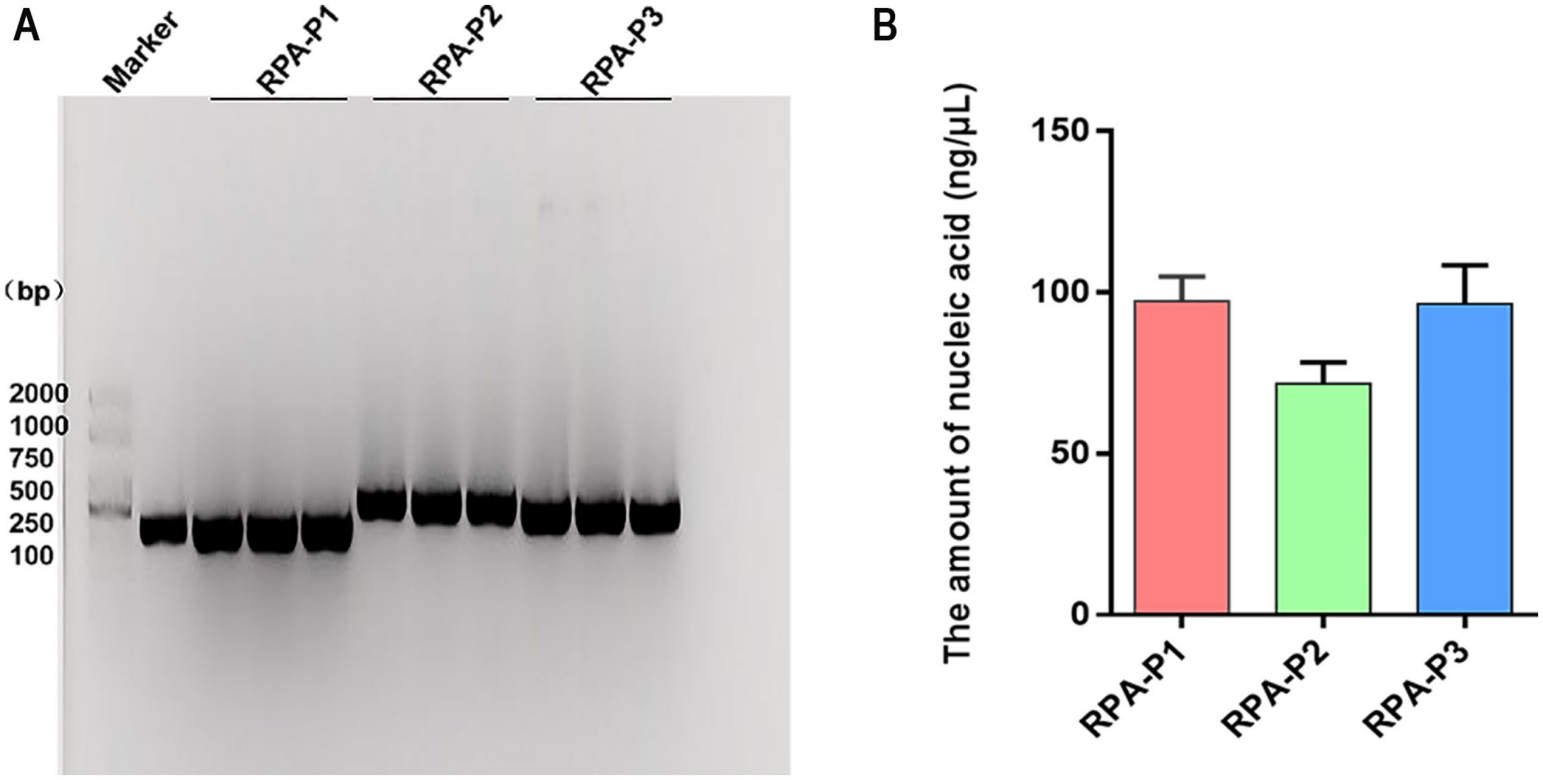
The amount of RPA amplification products were analyzed by Nucleic acid electrophoresis. **(A)** The amount of products amplified of RPA primers (RPA-P1, RPA-P2, RPA-P3) were analyzed by Nucleic acid electrophoresis. Maker: 2000 bp DNA Ladder Marker. Control: the positive sample control. **(B)** The amplification product yield were measured by RPA primers.

The amplification efficiency of three primer sets were systematically evaluated through multiple RPA amplification experiments coupled with 1% agarose gel electrophoresis analysis. All three RPA primer sets produced distinct, specific bands corresponding to the amplification products. Among these, the RPA-P1 and RPA-P3 primers generated higher product yields (Figure 2A). Gray value analysis further demonstrated that the RPA-P3 primer set exhibited the highest amplification product yield (Figure 2B). Based on the observed differences in amplification product yield, the RPA-P3 primer set was ultimately selected as the optimal primer for CPV RPA amplification.

### The influence of probe length on the binding of hybrid products

**Figure 3:**
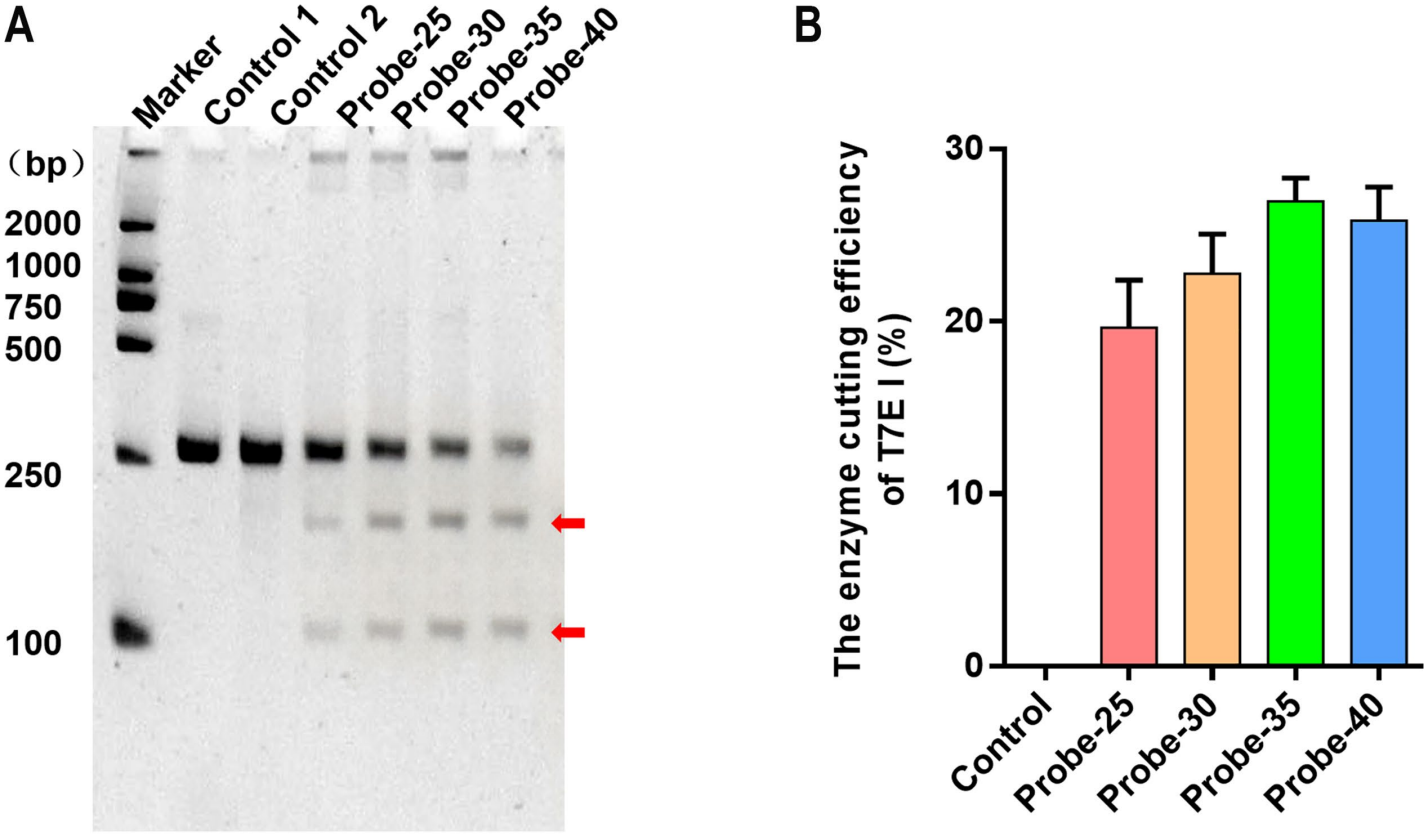
Different lengths of probes were hybridized with the VP2 gene for T7E I recognition and enzyme digestion. **(A)** The enzyme digestion levels of T7E I were determined by PAGE gel electrophoresis. Maker: 2000 bp DNA Ladder Marker. Control 1: the negative sample control (no probe and T7E I were added). Control 2: the negative sample control (no probe was added). The red arrows indicated bands cleaved by the enzyme. **(B)** The binding efficiency of the probes to the VP2 gene was calculated. Control: the negative sample control (no probe was added). Cleavage efficiency (%) = [Grayscale value of cut band/(Grayscale value of cut band+Grayscale value of uncut band)] × 100%, data represent means ± SD from three technical replicates.

In the screening of probe length reaction conditions, T7E I endonuclease digestion assays demonstrated that all four synthetic probes of varying lengths could bind to RPA products and be specifically recognized by T7E I, albeit with differing digestion efficiencies. Among them, Probe-35 exhibited the highest binding efficiency to RPA products, as evidenced by the distinct visualization of digestion products (Figure 3A). Quantitative grayscale analysis further confirmed that, under identical experimental conditions, probes with 35 and 40 base pairs (bp) outperformed those with 25 and 30 bp. Notably, Probe-35 achieved the highest digestion efficiency, indicating its superior binding capacity to RPA products (Figure 3B). Consequently, Probe-35 was selected as the optimal hybridization probe for subsequent experiments.

### The influence of probe concentration on the efficiency of enzymatic cleavage

**Figure 4:**
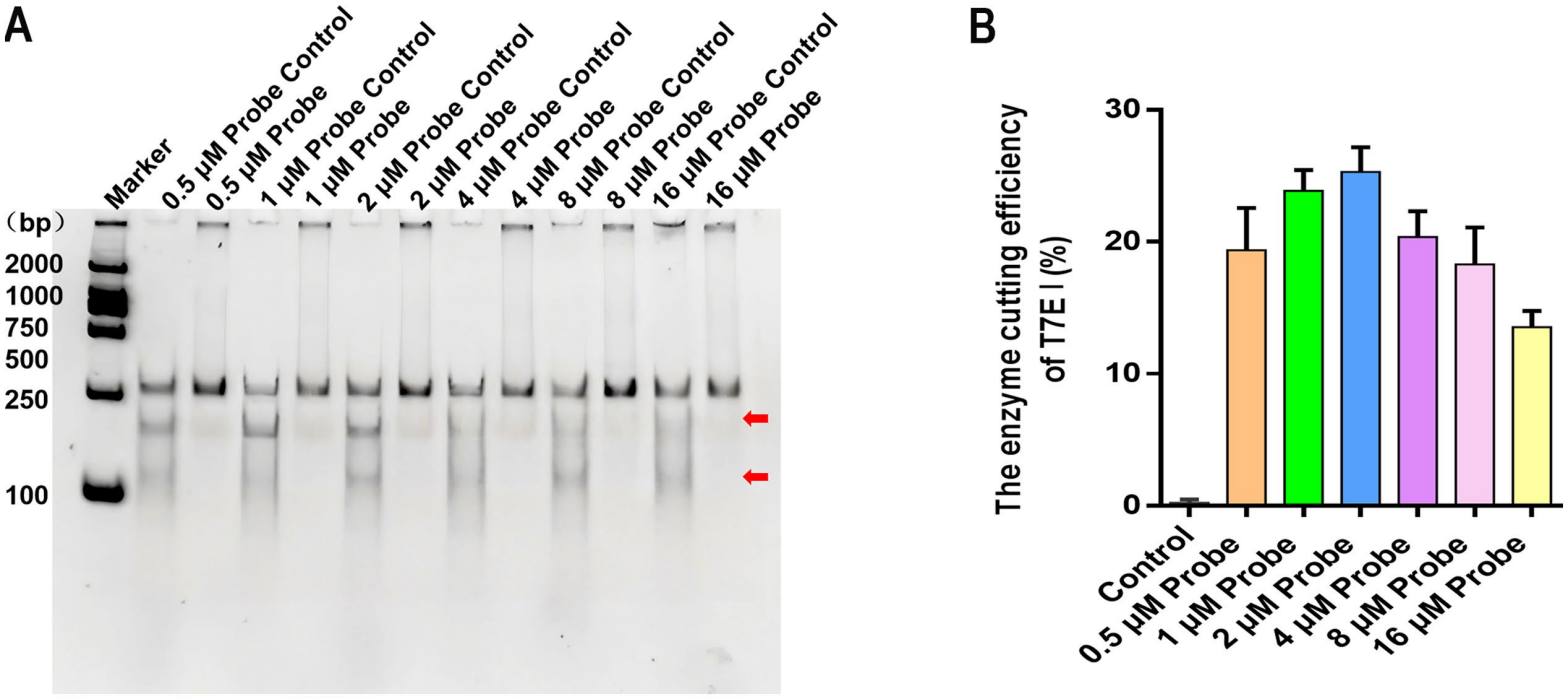
The effect of different probe concentrations on the formation of hybridization products and enzyme digestion efficiency. **(A)** The hybrid products were digested by different concentrations of probes (0.5 μM, 1 μM, 2 μM, 4 μM, 8 μM, and 16 μM). Maker: 2000 bp DNA Ladder Marker. Control: the negative sample control (no different concentrations of probes were added). **(B)** The effects of different concentrations of probes on the enzymatic digestion efficiency of T7E I were calculated. Control: the negative sample control (no probe was added). Cleavage efficiency (%) = [Grayscale value of cut band/(Grayscale value of cut band+Grayscale value of uncut band)] × 100%, data represent means ± SD from three technical replicates.

In the screening of probe concentration reaction conditions, the control without probe, a distinct band corresponding to the full-length target gene was observed at its expected size position, irrespective of the probe concentration. Crucially, no cleavage bands were detected, confirming the absence of non-specific digestion. In contrast, reactions containing T7E I and hybridized probe exhibited a clear cleavage pattern. Two distinct shorter bands appeared below the position of the full-length target gene across all probe concentrations tested (Figure 4A). Densitometric analysis of these results revealed that digestion efficiency varied with probe concentration. Specifically, efficiency initially increased but subsequently decreased as the probe concentration was raised. Maximum cleavage efficiency was achieved at a probe concentration of 2 μM, corresponding to approximately 60 ng of probe (Figure 4B). These results demonstrate that for 300 ng of RPA amplicon, hybridization efficiency between the target gene and the 35 bp probe, and consequently T7E I digestion efficiency, is optimal when using a 2 μM probe concentration.

### Optimization results of T7E I digesting concentration and time

**Figure 5:**
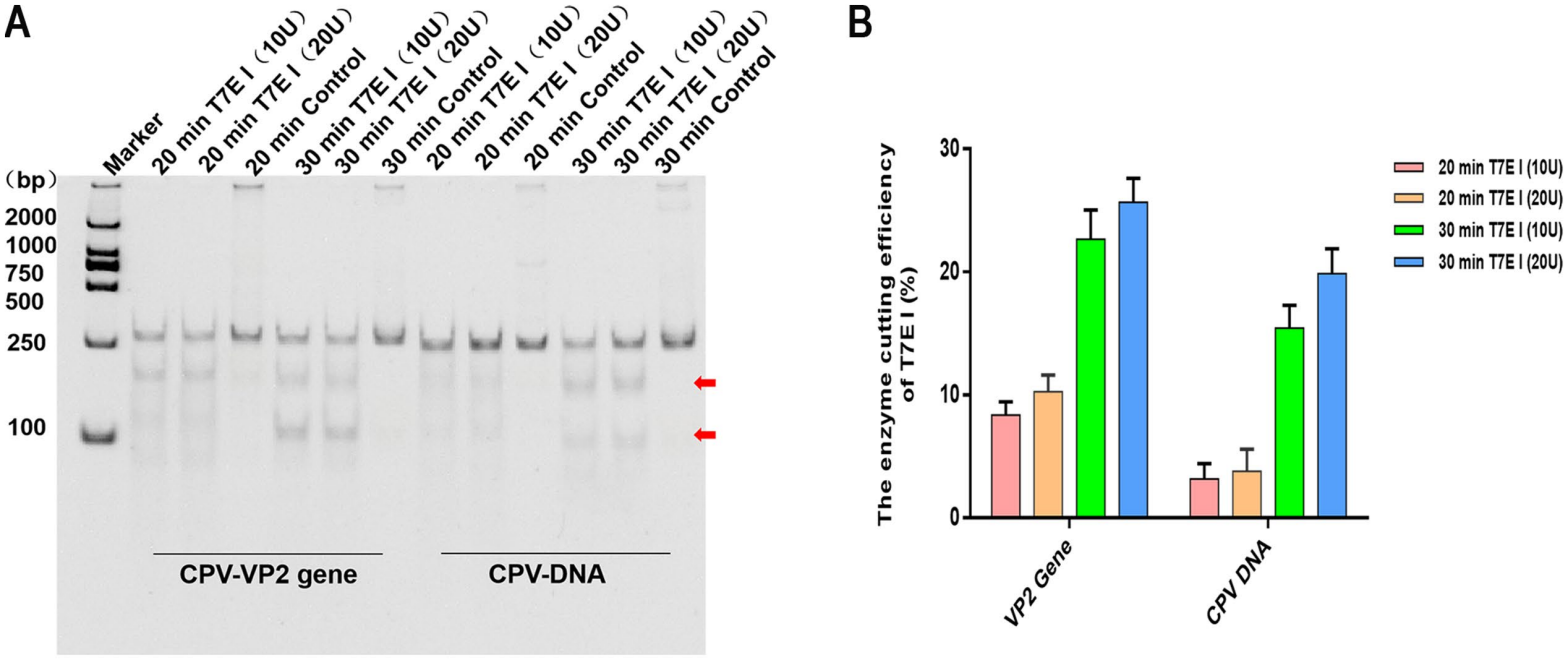
Effect of T7E 1 concentration and time on enzyme digesting efficiency. **(A)** The hybrid products of CPV VP2 or DNA were digested by different concentrations of T7E I (10 U and 20 U) for 20, 30 min. Maker: 2000 bp DNA Ladder Marker. Control: the negative sample control (no T7E I was added). The red arrows indicated bands cleaved by the enzyme. **(B)** The effects of different concentrations of T7E I concentration and time on the enzymatic digestion efficiency were calculated. Cleavage efficiency (%) = [Grayscale value of cut band/(Grayscale value of cut band+Grayscale value of uncut band)] × 100%, data represent means ± SD from three technical replicates.

In the screening of T7E I reaction conditions, the control without T7E I consistently exhibited distinct bands corresponding to the expected size of the target gene at the original position, regardless of incubation time. No smaller cleavage fragments were observed. Notably, in samples containing either the VP2 gene or CPV-DNA, the addition of T7E I to the reaction products resulted in the clear appearance of two smaller cleavage fragments below the target band (Figure 5A). Analysis of cleavage efficiency revealed that a T7E I concentration of 20 U yielded slightly higher efficiency compared to 10 U, although this difference was not statistically significant. Furthermore, extending the reaction time from 20 min to 30 min significantly enhanced the cleavage efficiency (Figure 5B). These results demonstrate that the optimal conditions for maximal efficiency involve cleavage with 20 U of T7E I for 30 min.

### End-point visualization by NMED assay

**Figure 6:**
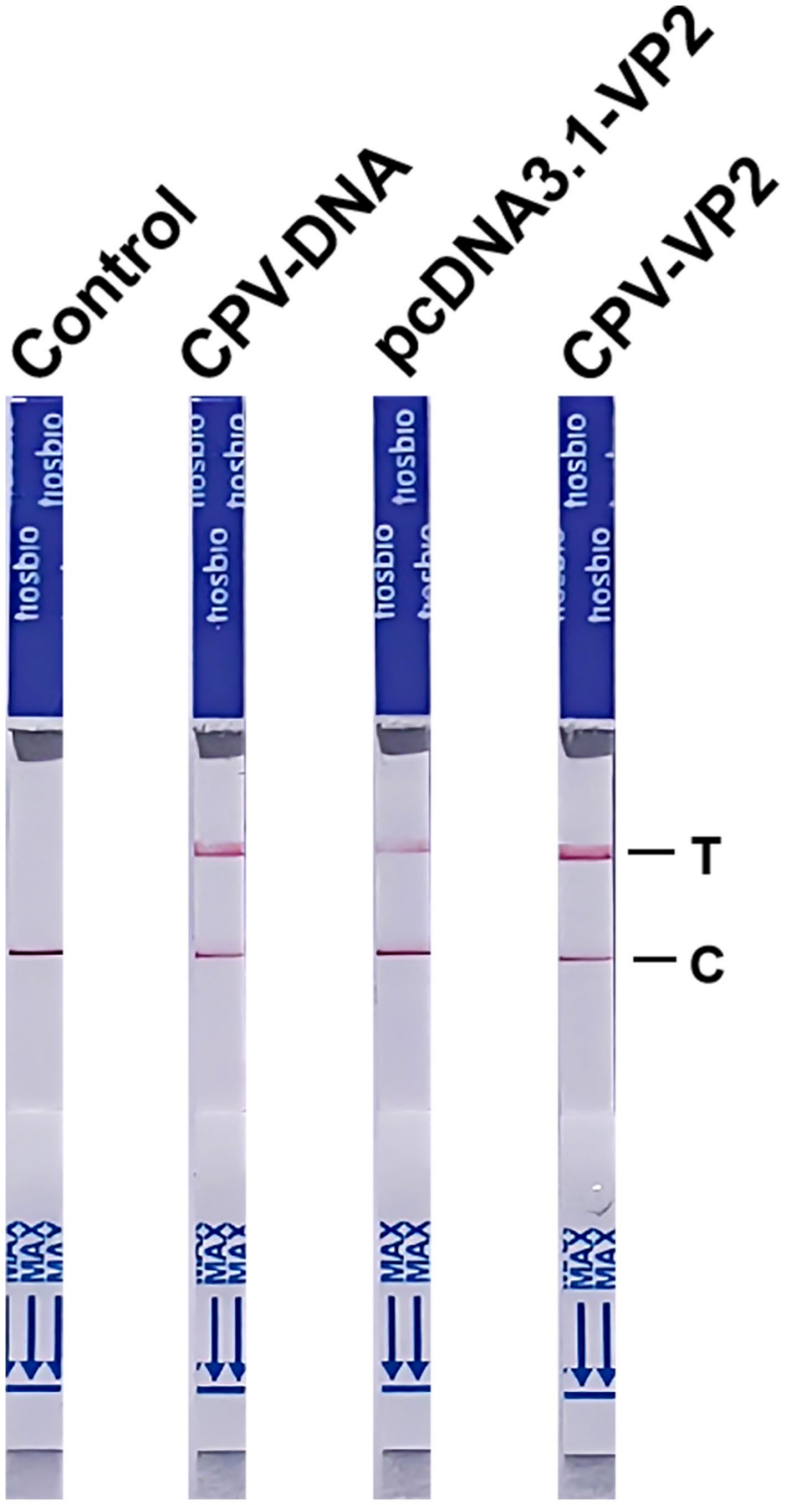
The NMED results for different samples. Control: the sample was nuclease-free water; CPV-DNA: the sample was the DNA of CPV; pcDNA3.1-VP2: the sample was the plasmid pcDNA3.1-VP2; CPV-VP2: the sample was the VP2 gene of CPV. C line: the control line; T line: the test line.

Under optimized reaction conditions, we subjected the plasmid pcDNA3.1-VP2, CPV-DNA, and the CPV VP2 gene to experimental testing. The results were detected using LFA and the LFA results showed the control was negative, while all three genetic samples tested positive. The reading time was short, with results obtainable within 10 min (Figure 6). These results demonstrate the feasibility of end-point visualization for CPV detection based on the NMED assay.

### Specificity of the NMED assay

**Figure 7:**
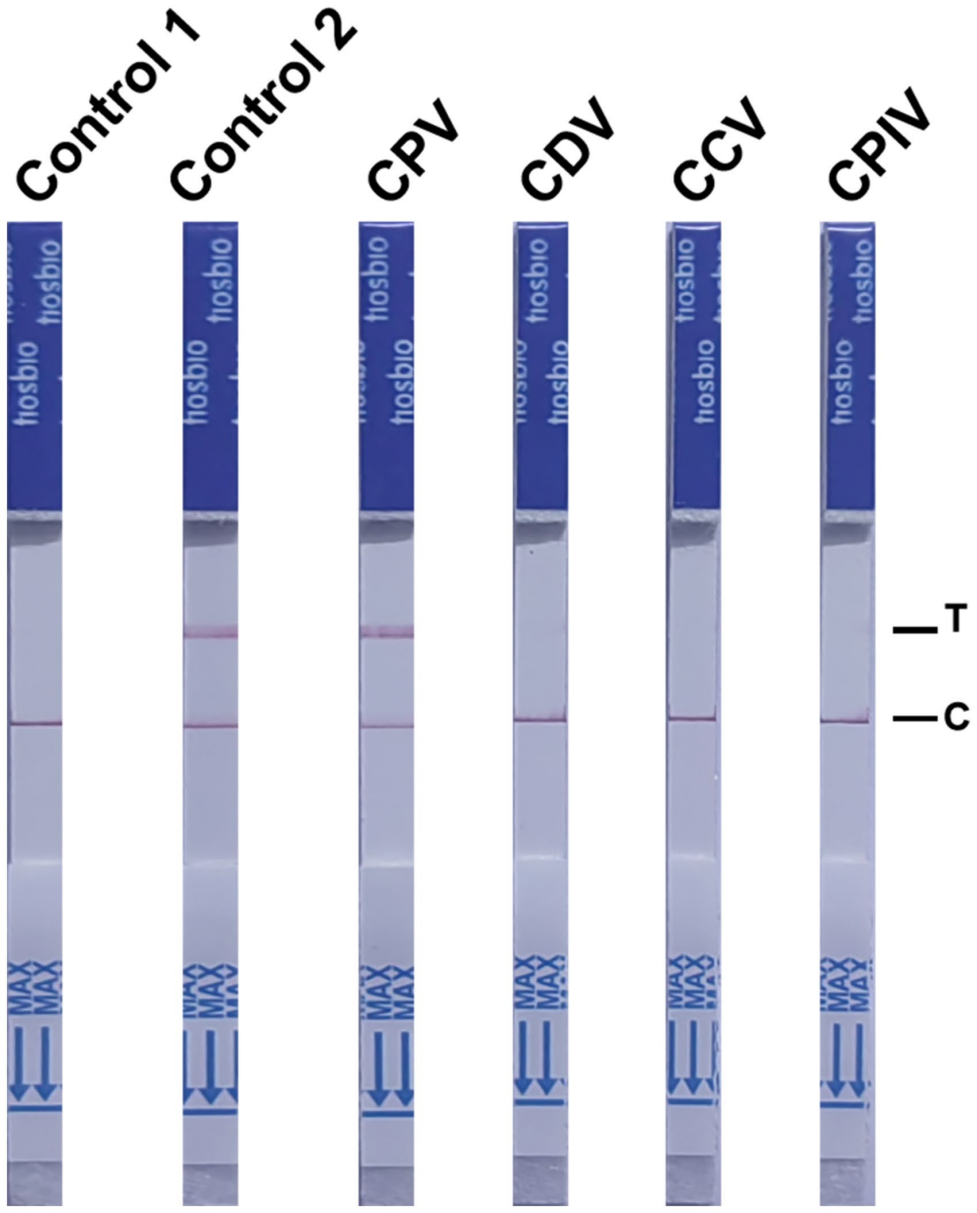
The NMED results of different types of virus samples. Control 1: the sample was nuclease-free water; Control 2: the sample was the DNA of CPV; CPV: the sample was the virus of CPV; CDV: the sample was the virus of CDV; CCV: the sample was the virus of CCV; CPIV: the sample was the virus of CPIV. C line: the control line; T line: the test line.

To evaluate the specificity of the NMED assay for detecting CPV, NMED testing was performed on samples containing CPV, CDV, CPIV, CAV-1, and CCV. The LFA revealed negative results for the control, while positive results were obtained for the detection of the viral VP2 gene. Differential detection outcomes were observed across the viruses tested: only the CPV strip yielded a positive result, whereas the strips for CDV, CPIV, CAV-1, and CCV all displayed negative results (Figure 7). These findings indicate that the NMED assay exhibits high specificity, showing no cross-reactivity with other common canine viruses.

### Sensitivity of the NMED assay

**Figure 8:**
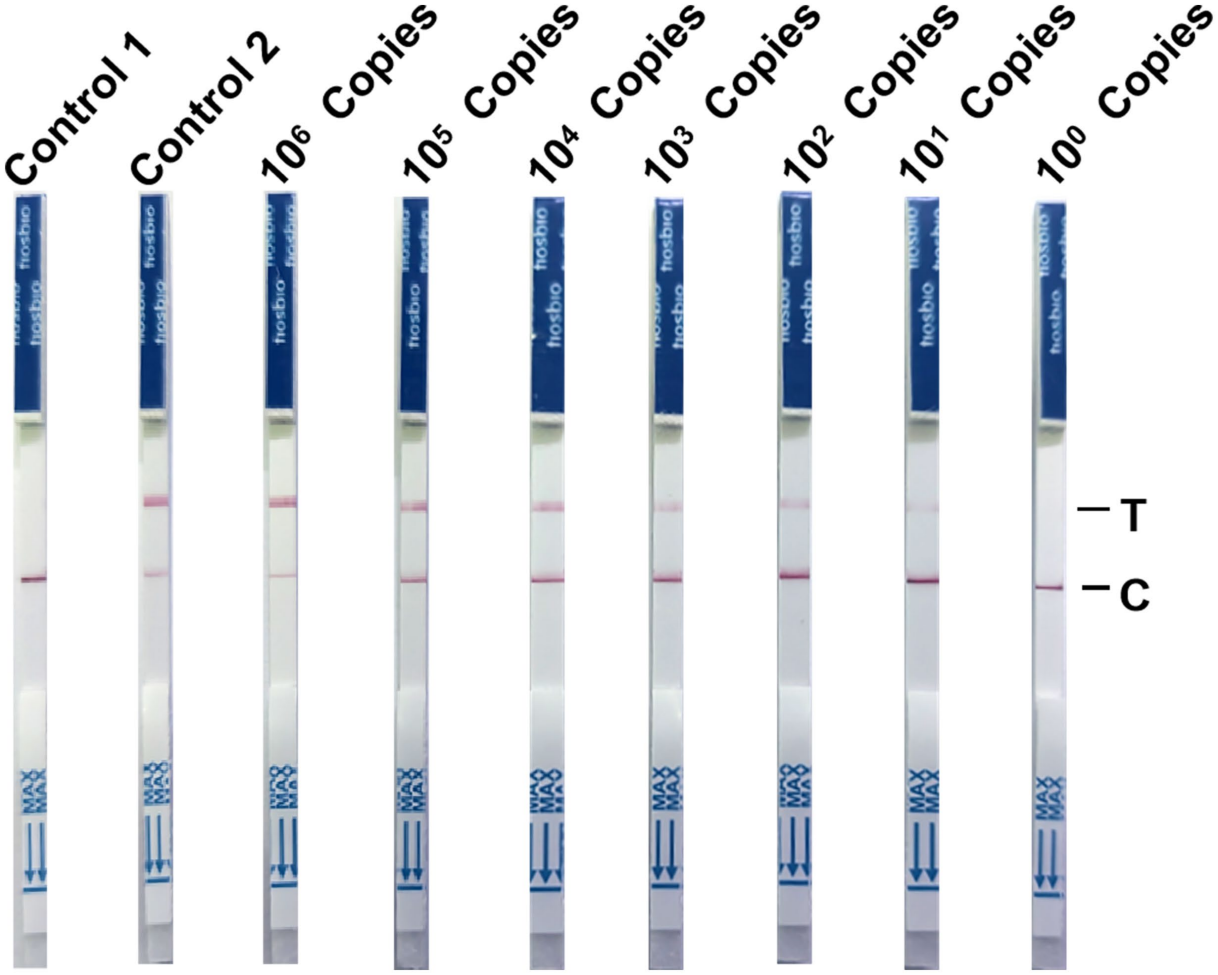
The NMED results of virus gradient copy number samples. Control 1: the sample was nuclease-free water; Control 2: the sample was the DNA of CPV; 10^6^ Copies: the sample was 10^6^ viruses; 10^5^ copies: the sample was 10^5^ viruses; 10^4^ copies: the sample was 10^4^ viruses; 10^3^ copies: the sample was 10^3^ viruses; 10^2^ copies: the sample was 10^2^ viruses; 10^1^ copies: the sample was 10^1^ viruses; 10^0^ copies: the sample was 10^0^ viruses. C line: the control line; T line: the test line.

To investigate the sensitivity of the NMED assay for CPV detection, we utilized NMED to test serially 10-fold diluted pcDNA3.1-VP2 plasmids, spanning a concentration range from 10^6^ to 10^0^ copies per reaction. The results demonstrated that the control yielded negative results, whereas the viral VP2 gene was consistently detected as positive. As the plasmid concentration gradually decreased, the detection results transitioned progressively from positive to negative. At a plasmid concentration of 10^1^, LFA still yielded a positive result. However, when the plasmid concentration reached 10^0^, the assay yielded a negative result. The reading time was short, with results obtainable within 10 min (Figure 8). These findings collectively indicate that the NMED method can achieves a detection limit of greater than 10^1^ copies for the target virus, thereby demonstrating high sensitivity.

### Validation of the NMED assay for clinical isolates

**Figure 9:**
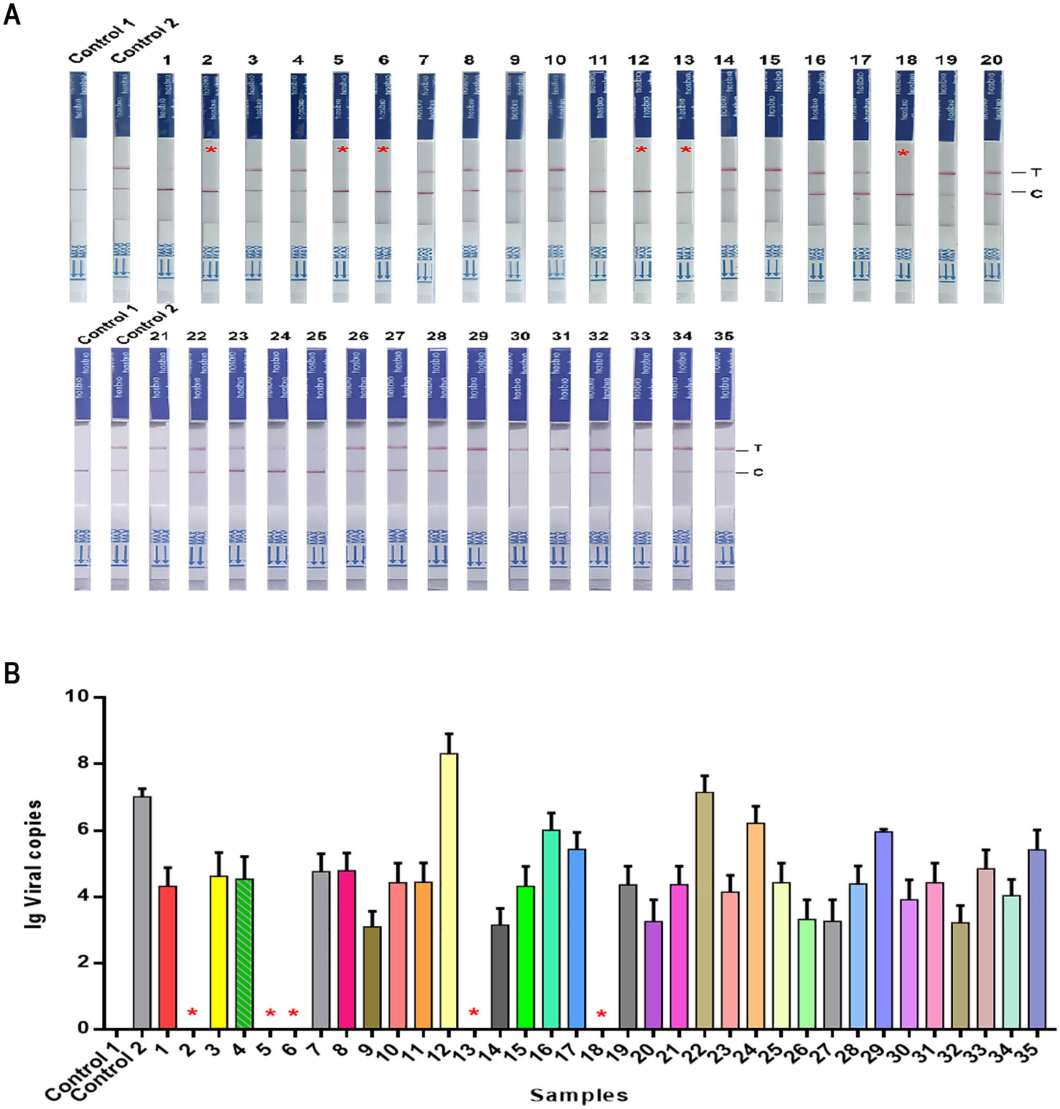
The detection results of CPV clinical samples. **(A,B)** Control 1: the sample was nuclease-free water; Control 2: the sample was the DNA of CPV; 1 ~ 35: different CPV clinical samples. *The negative sample.

We also evaluated the performance of NMED in diagnosing CPV using clinical isolates. 35 suspected CPV-positive fecal samples, initially screened as positive by LFA and collected from veterinary hospitals, were subjected to analysis. Using qPCR as the gold standard, the clinical detection capability of NMED was compared. NMED detection identified 6 negative results and 29 positive results (Figure 9A), corresponding to a positivity rate of 82.86% (Table 2). In contrast, qPCR analysis revealed that 5 of the 35 viral samples yielded negative results, while 30 were positive (Figure 9B). Compared with the qPCR gold standard, the NMED demonstrated a concordance rate of 97.14% (Table 2). These results demonstrate that the NMED method is reliable and exhibits significant potential for application in clinical diagnostics.

**Table 2:** Clinical validation of NMED assay for the CPV.

		NMED			Coincidence Rate (CR)
		Positive	Negative	Total	
qPCR	Positive	29	1	30	97.14%
	Negative	0	5	5	
	Total	29	6	35	
LFA	Positive	29	6	35	82.86%
	Negative	0	0	0	
	Total	29	6	35	

35 clinical isolates were used to evaluate the NMED assay. Coincidence Rate (CR) = 100% × [(both positive + both negative)/total samples]; qPCR was the standard TaqMan probe method for CPV. LFA was the lateral flow assay for CPV at the veterinary hospitals.

## Discussion

CPV is a globally prevalent, highly lethal pathogen causing infectious disease in dogs, posing a particularly severe threat to puppies. The virus undergoes antigenic drift in its capsid protein, increasing the risk that variant strains may evade the protection offered by existing vaccines. Early diagnosis is critical for reducing mortality rates in puppies (from 73.4 to 28.9%), lowering per-dog treatment costs, and is essential for effective disease management and outbreak control (27). Traditional CPV detection technologies, however, face significant challenges in practical application. Classical methods, represented by virus culture, morphological observation, and biochemical identification, typically require long detection cycles of 3–7 days. They also suffer from inherent limitations such as poor specificity (cross-reactivity rate >15%) and low sensitivity (limit of detection >10^3^ CFU/mL) (25). Although real-time quantitative polymerase chain reaction (RT-qPCR), as the “gold standard” for nucleic acid testing, achieves significantly higher sensitivity at levels of 10 copies/μL, it presents other drawbacks. Its requirement for precision temperature-control equipment leads to high per-test costs, its amplification cycle remains relatively lengthy, and it carries a significant risk of aerosol contamination. Immunochromatographic assays (colloidal gold lateral flow tests), while enabling rapid detection within 15 min, exhibit 1–2 orders of magnitude lower sensitivity than RT-qPCR (limit of detection >10^4^ copies/μL). Additionally, these assays demonstrate a false-positive rate of 5–10% and are fundamentally incapable of providing quantitative results (26).

In recent years, breakthrough advancements in isothermal nucleic acid amplification technologies have ushered in a new era for POCT. Technical systems based on LAMP and RPA enable target amplification within 30 min under constant temperatures ranging from 38 °C to 45 °C, boasting high detection sensitivity comparable to that of RT-qPCR. The real-time RPA detection system for CPV-2 developed by the Geng team not only achieved a 100% clinical concordance rate but also pushed the LOD down to 10 copies/μL without cross-reactivity. However, this technology still faces technical bottlenecks, including a relatively high false-positive rate (approximately 3–5%) and a complex reaction system involving 8–10 enzyme components (27). Wang et al. developed a lateral flow dipstick-RPA combined technology, which, through the synergistic effect of specific primer-probe sets and nucleic acid test strips, enabled the detection of FPV at 10^2^ copies/μL within 15 min at 38 °C, representing a 10-fold increase in sensitivity compared to conventional PCR (28). Of greater significance is the integration of isothermal amplification technologies with CRISPR-Dx systems. Leveraging the collateral cleavage activity of certain Cas proteins, these integrated systems meet POCT requirements through methods such as lateral flow strips, reaction solution color changes, and electrochemical biosensors. The RPA-Cas12a fluorescent detection system developed by the Wang team allowed FPV identification within 25 min, achieving an LOD of 1 copy/μL with 100% concordance to qPCR results and demonstrating excellent specificity (29). Similarly, the ERA-Cas12a platform constructed by Wei et al. eliminated reliance on traditional instrumentation, enabling visual interpretation of PPV infection under 37 °C isothermal conditions with a sensitivity of 3.75 × 10^2^ copies/μL and precise differentiation from other swine pathogens (30). Despite the rapid development of CRISPR diagnostic technologies, critical bottlenecks hinder their clinical application. For instance, the requirement for Protospacer Adjacent Motif (PAM) recognition significantly constrains the design flexibility of crRNA. Additionally, non-specific trans-cleavage activity of Cas proteins may lead to false-positive results, and insufficient stability of single-stranded DNA cleavage activity compromises detection reproducibility. Therefore, developing novel detection systems or improving existing technologies is crucial to ensuring the reliability and clinical applicability of viral detection. In the context of EMC, Jalamilou et al. utilized the digestion characteristic of mismatch-specific endonucleases to observe whether mutations existed in the rpoB, inhA, katG, gyrA, and rrs genes from multidrug-resistant/pan-drug-resistant *Mycobacterium tuberculosis* (MTB) strains. This technique enhanced the detection capability for multidrug-resistant and pan-drug-resistant MTB, and had the advantages of eliminating expensive equipment, customizable mutation targeting, and being rapid and efficient (31). In a parallel advancement, Chowdhury P et al. developed a simple electrochemical biosensor based on differential pulse voltammetry (DPV) for the specific detection of KRAS mutations. The sensor operates on the principle that T7E I recognizes and cleaves mismatched sites generated by KRAS gene mutations, thereby removing the 5′-biotin moiety from capture probes. This cleavage event results in a reduced DPV signal compared to wild-type sequences. The sensor exhibits a low detection limit of 11.89 fM, and the ability to distinguish 0.1% mutant alleles from wild-type DNA. These findings confirm its potential application in detecting mutations for early disease diagnosis (32).

In this study, a novel nucleic acid detection method, named NMED (Nucleic Acid Detection based on the synergy of specific fluorescent probes and T7E I recognition), was innovatively proposed, and a rapid detection system for CPV was successfully established. Experimental data demonstrated that this method specifically targets CPV with no cross-reactivity with other viruses, exhibiting high specificity. The detection sensitivity reached over 10 copies/μL, highlighting its robust analytical performance. Notably, the integration of EMC with colloidal gold immunochromatography LFA not only enhanced the detection sensitivity of RPA but also reduced the risk of false positives, addressing a critical challenge in nucleic acid amplification-based diagnostics.

In clinical validation, the NMED assay was applied to 35 clinical samples, showing a detection consistency of 97.14% compared with qPCR, confirming its reliability in practical settings. Looking forward, by optimizing the probe labeling system and integrating automated reading devices, this method is expected to promote the in-depth application of POCT technology in veterinary clinics, enabling rapid and on-site diagnosis. The successful development of NMED not only provides a novel solution for clinical CPV detection but also exhibits broad applicability beyond CPV. Its detection principle can be extended to the rapid detection of other Parvoviridae viruses, such as Feline Panleukopenia Virus (FPV) and Porcine Circovirus (PCV), thereby demonstrating significant industrialization potential. This advancement not only enriches the toolkit for veterinary virology diagnostics but also paves the way for the development of versatile, high-performance detection platforms for a range of pathogens.

## Data Availability

The original contributions presented in the study are included in the article/supplementary material, further inquiries can be directed to the corresponding author/s.
